# Targeted literature review of the global burden of gastric cancer

**DOI:** 10.3332/ecancer.2018.883

**Published:** 2018-11-26

**Authors:** Montserrat Casamayor, Robert Morlock, Hiroshi Maeda, Jaffer Ajani

**Affiliations:** 1 IQVIA, C/Provença 392-3ª, 08025 Barcelona, Spain; 2 Astellas Pharma Global Development, Inc., 1 Astellas Way, Northbrook, IL 60062 USA; 3 University of Texas MD Anderson Cancer Center, 1515 Holcombe Blvd, Houston, TX 77030 USA

**Keywords:** gastric cancer, gastroesophageal junction, humanistic, economic, burden, global

## Abstract

Gastric cancer (GC) and gastroesophageal junction cancers (GEJCs) are the third leading cause of cancer-related death worldwide. Although several studies have evaluated the epidemiology and management of GC and GEJC, to our knowledge, no global estimates of the economic burden of GC and GEJC have yet been reported. This targeted literature review was conducted to summarise the epidemiology and management of GC and GEJC and to estimate its global economic and humanistic burden.

The incidence of GC and GEJC is highest in Eastern Asia, several South and Central American countries and Central and Eastern Europe and lowest in North America and Africa. Prognosis is generally poor; the global 5-year survival rate is 5%–10% in advanced stages. Patients with GC and GEJC have more severe symptoms compared with patients with other cancers, and health-related quality of life (HRQoL) worsens as the disease progresses. Given the rapid progression of GC and GEJC at advanced stages, chemotherapy, despite its toxicity, improves HRQoL compared with best supportive care.

The costs of GC/GEJC are generally higher than for other cancers; in the US, the average annual cost per patient between 1998 and 2003 was 46,501 USD, compared with 29,609 USD and 35,672 USD for colorectal and lung cancer, respectively. Based on the 2012 incidence data and average costs per patient, estimates of the annual financial burden of GC and GEJC revealed great regional differences. Japan and Iran had the highest (8,492 million USD) and lowest (27 million USD) costs for 2017, respectively, while the estimate for the US was 3,171 million USD. The overall annual cost of GC and GEJC estimated for 2017 in a geographic area including Europe (France, Germany, Italy, Spain and the UK), Asia (Iran, Japan and China), North America (Canada and the US) and Australia was 20.6 billion USD.

## Background

Gastric cancer (GC) is an aggressive cancer that develops in the stomach and represents one of the leading overall causes of deaths worldwide, ranking fifth in cancer incidence and third in cancer-related deaths [1]. GC can develop in any part of the stomach; however, the majority of GCs are located in the pyloric area. Gastroesophageal junction cancers (GEJCs) develop in the gastroesophageal junction (GEJ) and can be classified as GC or oesophageal cancer depending on their extension in the stomach [2]. The American Joint Committee on Cancer considers all GEJCs to be oesophageal unless they arise in the stomach >5 cm from the GEJ [2]; however, GEJCs are often referred to as GC in the literature.

It is estimated that 80%–90% of GCs are adenocarcinomas and can be classified based on their histological characteristics as differentiated (intestinal) or undifferentiated (diffuse) type (Lauren classification) [3]. The differentiated type has a relative occurrence of approximately 54%, is more common in elderly males, and develops slowly, whereas the diffuse type is more common in females of a younger age and has a worse prognosis [4]. Similar to GC, most GEJCs (90%) are also adenocarcinomas while the remainder are classified as either squamous cell carcinomas or unspecified carcinomas [5].

Worldwide, the incidence of GC varies widely. The highest age standardised-incidence rate (ASIR) per 100,000 individuals is observed in Eastern Asia, particularly in China, Mongolia, Japan and Republic of Korea [6] (35.4 males; 13.8 females), Central and Eastern Europe (20.3 males; 8.9 females) and South America (14.2 males; 7.0 females), particularly concentrated in the Pacific regions of South America [7, 8]. The lowest incidence is observed in North America (5.5 males; 2.8 females) and Africa (4.5 males; 3.2 females) [7]. The global incidence has declined over the last decades, and estimates up to the year 2030 represent a projected 2.3% annual decrease [6].

Early stages of GC typically present with minimal or no symptoms; therefore, GC is frequently diagnosed at advanced stages, resulting in poor prognosis [9]. This is particularly evident in Western countries where, due to the low incidence of GC, awareness is low and screening programmes are not established [9]. In contrast, in some Asian countries (i.e. Republic of Korea and Japan) where the incidence is higher and where mass screening programmes are cost-effective and widely available, early detection occurs more frequently [9–11]. This evidence suggests that early diagnosis represents an important strategy to improve outcome and survival.

Several genetic factors, as well as age, sex, family history, radiation exposure, Helicobacter pylori (H. pylori) infection and smoking, were all shown to be associated with an increased risk of GC [6, 12–14] and it is likely that differences in the geographical distribution of risk factors may be partly responsible for the observed variation in incidence [6]. Among the potential prognostic and therapeutic biomarkers for GC, HER2 is the only biomarker currently screened in clinical practice and is used to identify patients who will respond to trastuzumab; overexpression of HER2 occurs in 9%–38% of GC and appears to impact gastric carcinogenesis [15]. Another potential therapeutic biomarker is PD-L1/2 (ligands of programmed death receptor 1 [PD-1]). Since its expression is elevated in Epstein-Barr virus (EBV)-positive tumours, PD-L1/2 antagonists may be effective in the treatment of EBV-related GCs. However, the validity of PD-L1/2 as a predictive biomarker has not been fully established [15]. Exploratory biomarkers include Claudin 18.2 [16], tumour mutation burden, including microsatellite instability [15], and gamma interferon [17]. Further understanding and identification of predictive biomarkers for GC will help improve outcomes.

Despite the increased awareness of GC and the growing effort to develop new therapies, and although several studies have investigated the economic cost of GC [18–45], to our knowledge, no global estimates of the economic burden of GC and GEJC had been reported at the time we began writing this manuscript. This work summarises the current landscape of GC and GEJC described in the literature, provides an overview of its epidemiology and management and estimates its global economic and humanistic burden.

## Methods

To evaluate the burden associated with GC and GEJC, four targeted literature reviews were conducted to identify the following outcomes in 1) Epidemiology: incidence, prevalence, morbidity, mortality, trend of incidence and trend of prevalence; 2) Management: current management and availability of national/international treatment guidelines; 3) Humanistic burden: patients’ HRQoL, disease- versus treatment-related impact on HRQoL and changes of humanistic burden after treatment implementation and 4) Economic burden: direct/indirect cost in different regions, cost trends after treatment implementation, health resource utilisation and management of treatment.

### Literature searches

Databases that were searched include PubMed, Cochrane Library (Cochrane Database of Systematic Reviews, Cochrane Central Register of Controlled Trials, Database of Abstracts of Reviews of Effects, Health Technology Assessment [HTA] and National Health Service Economic Evaluation Database), Cost-Effectiveness Analysis Registry, EconLit and IQVIA proprietary HTA Accelerator. Proceedings of international scientific meetings (2014–2017) included American Society of Clinical Oncology, American Society of Clinical Oncology-Gastrointestinal Cancers Symposium, European Society for Medical Oncology, European Cancer Organisation, International Gastric Cancer Congress, Gastrointestinal Cancers Symposium and International Society for Pharmacoeconomics and Outcomes Research. Websites of the national cancer associations and Google searches were used to identify the most recent epidemiology data and guidelines. The search strategy applied to all databases included disease-specific keywords (e.g. ‘stomach neoplasms’, ‘gastroesophageal’ and ‘tumour’) and keywords associated with epidemiology endpoints (e.g. ‘incidence’, ‘prevalence’ and ‘mortality’), management endpoints (e.g. ‘guidelines’ and ‘real world’), humanistic endpoints (e.g. ‘quality of life’, ‘health status’ and ‘humanistic burden’) and economic endpoints (e.g. ‘cost’, ‘healthcare cost’ and ‘cost of illness’). The search was limited to studies published within the last 15 years. No geographic limits were applied; however, the priority was given to studies that focused on more than one country and on key geographic areas. No language restriction was applied to the search but only studies with an abstract written in English and full text in English, German, French, Italian or Spanish were eligible for inclusion.

### Study eligibility criteria

The reviews were conducted in accordance with the guidelines from the Centre for Reviews and Dissemination [46] and NICE guidance [47] and were based on the PICOS (P: population, I: intervention, C: comparator, O: outcomes and S: study design) criteria. The population of interest was adults with GC or GEJC regardless of stage, histology or biomarker. No restrictions with respect to intervention or comparator were applied.

### Study selection

All retrieved articles were reviewed by a researcher, and those considered irrelevant were removed. The remaining articles were further assessed to identify those studies that met the eligibility criteria. A quality check was conducted on a sample of the selected articles/abstracts by a second researcher, and a full-text review was conducted to determine relevance to the eligibility criteria. A quality assessment was conducted on all selected studies by one reviewer, and a second reviewer performed a further quality assessment on a sample of all the studies.

Based on the data available in the reviewed articles, we calculated the annual cost of GC and GEJC in different geographic regions and estimated an annual global financial cost expected in 2017.

## Results

**Figure 1.**
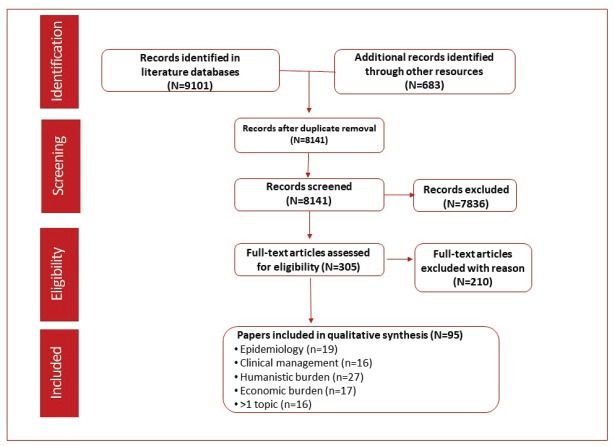
PRISMA flow diagram of studies identified through the predefined search strategy.

The literature search identified 9,101 records through the selected databases and 638 records using other resources (congresses, websites and proprietary HTA accelerator database). After duplicates were removed, 8,141 records were screened and 7,836 were excluded based on the eligibility criteria. A full-text analysis was conducted on the remaining 305 articles and resulted in the exclusion of 210 articles that did not meet the eligibility criteria. A total of 95 references were included in the qualitative analysis. Epidemiology outcomes were reported in 19 records, clinical management in 16, humanistic outcomes in 27 and economic outcomes in 17. More than one outcome was reported in 16 articles (Figure 1).

### Epidemiology

**Figure 2.**
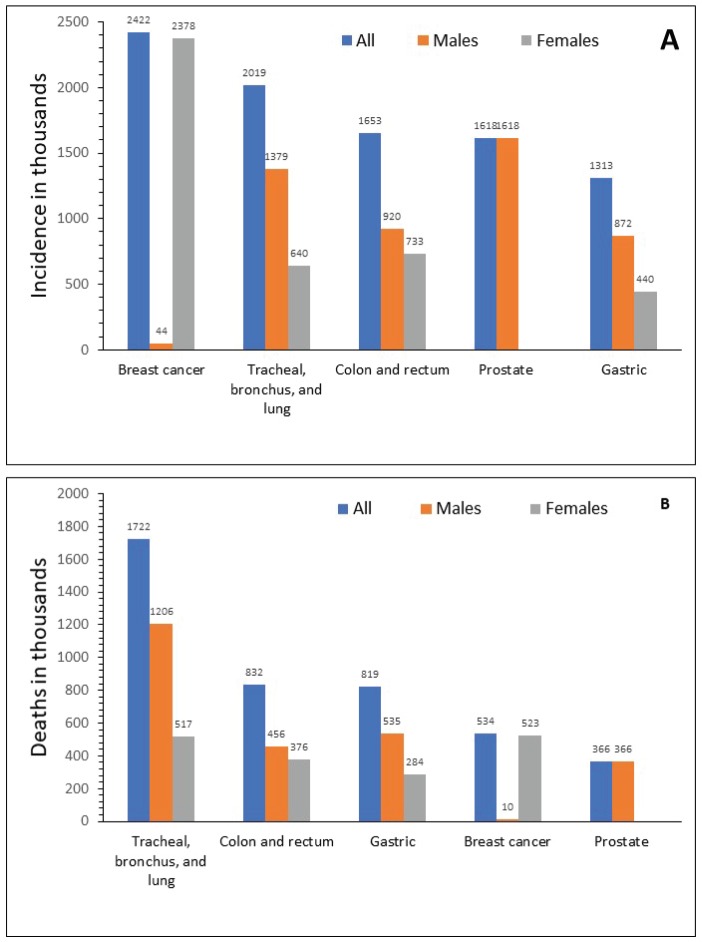
Global incidence (a) and mortality (b) for different types of cancer in 2015. Source: Fitzmaurice 2017 [1].

In 2015, GC ranked fifth for incidence in both sexes after breast, lung, colorectal and prostate cancer (Figure 2a), and third for cancer-related mortality in both sexes after lung and colorectal cancers (Figure 2b) [1]. In 2012, the global ASIR was 12.1 per 100,000 individuals, ranging between 10.6 and 12.7 per 100,000 for more and less developed regions, respectively [7, 48]. The highest ASIR (per 100,000) of GC was observed in Eastern Asia (overall, 24.2; China, 22.7; Mongolia, 32.5; Japan, 29.9; Democratic People’s Republic of Korea, 14.3; and Republic of Korea, 41.8), Central and Eastern Europe (13.5) (range 10.3─24.2) and South America (10.3), particularly concentrated in the Pacific regions of South America (i.e. Colombia, Ecuador, Peru and Chile) [7, 8]. The lowest incidence was observed in North America (4), Africa (3.8) and the Eastern Mediterranean region (5.5) [7, 49]. Regardless of the geographic region, incidence was 2–3 times higher in men than in women, and higher in black and Latino populations compared with non-Hispanic white populations [13].

Mortality associated with GC was 2–3 times higher in men than women [1]; the highest age-standardised mortality rates (per 100,000) were reported in East Asia (16.5), Central and Eastern Europe (10.9) and South America (8.5), while the lowest rate occurred in North America (2.1) [6, 12]. Over the last two decades, a steady decline in mortality rates in patients with GC was observed in both developed and developing countries [1, 6, 50, 51].

Adenocarcinomas represent the most common histological type of GC (80%–90%), and the ‘differentiated’ type is the most frequently observed [3, 4].

In 2012, among all GCs reported worldwide, 27% were cardia and 73% were non-cardia located; the pyloric area was the most reported location for non-cardia GC [52]. The majority of both cardia (59%) and non-cardia (63%) GC cases occurred in Eastern/Southeastern Asia and more than 50% of both cardia and non-cardia GC cases were reported in China [52].

Our search indicates that the prognosis of GC was poor in all geographical areas; the global overall 5-year survival rate was 20%–30% and 5%–10% in the advanced stage (stages III and IV, respectively) [53–55].

### Management

For resectable GCs, the guideline recommendations from the European Society for Medical Oncology [3], the Spanish Society of Medical Oncology [56] and the Japanese Gastric Cancer Association [57] all include endoscopic resection at early stages. For localised GC, a multidisciplinary approach including gastrectomy and postsurgical (adjuvant) [3, 10, 56–58] or perisurgical (before and after) [3, 10, 56, 58] chemotherapy with [3, 10, 56, 58] or without [57] radiation, is recommended. For patients with stage II or III GC undergoing tumour resection/lymphadenectomy D2, the Italian Association of Medical Oncology recommends adjuvant chemotherapy (monochemotherapy with fluoropyrimidine or chemotherapy regimens based on capecitabine or oxaliplatin) [58]; whereas perisurgical chemotherapy is recommended by the European Society for Medical Oncology guidelines for stage IV or higher resectable GC [3] and by the Italian Association of Medical Oncology guidelines for locally advanced GC (T3 or clinical N+) [58] and fluoropyrimidine or platinum derivatives are recommended for perisurgical chemotherapy [34, 58]. FLOT (i.e. Fluorouracil, leucovorin, oxaliplatin and docetaxel) represents a new promising perioperative regimen option for patients with locally advanced, resectable GC [59]. Post-surgery chemotherapy with S-1 monotherapy (tegafur, gimeracil and oteracil potassium), capecitabine plus oxaliplatin or S-1 plus oxaliplatin is recommended by the Japanese Gastric Cancer Association guidelines [57].

Six guidelines (Europe, Italy, Japan, Spain, UK and the US) provide recommendations for first-line therapy for advanced and metastatic GC and GEJC [3, 47, 56–58, 60]. Chemotherapy is the recommended first-line therapy for advanced GC and GEJC but there is no consensus among the available guidelines on the specific regimen that should be used (Table S1 and S2 in Supplement). The US [60] and Japanese [57] guidelines recommend doublet over triplet chemotherapy to reduce toxicity and indicate that triplet therapy be used only in fit patients who have frequent access to toxicity screenings. The US guidelines recommend a regimen with a 5-Fluorouracil (5-FU) or capecitabine and either cisplatin or oxaliplatin, whereas the Japanese guidelines recommend a regimen of S-1 and cisplatin as the first choice, followed by the combination of capecitabine and cisplatin. The European guidelines [3] recommend both doublet and triplet protocols, and favour EOX (epirubicin, oxaliplatin and capecitabine) over ECF (epirubicin, cisplatin and fluorouracil) and capecitabine over infused 5-FU due to better overall survival. For the management of HER2-positive GC and GEJC, all guidelines recommend adding trastuzumab to first-line chemotherapy regimens, which commonly include cisplatin and a fluoropyrimidine [56, 58, 60]. Second lines of chemotherapy include the same regimens for GC and GEJC and are generally based on the patients’ condition. The preferred regimen for second-line treatment is ramucirumab plus paclitaxel, or ramucirumab monotherapy if the patient is not eligible for paclitaxel (Table S2 in Supplement) [3, 10, 56–58]. A PD-1 inhibitor has recently been approved as third-line or higher therapy for GC/GEJC patients in the US [61].

Evaluation of the reports of advanced stage GC and GEJC management in clinical practice shows a lack of consensus on the choice of chemotherapy regimens. In 2016, the most commonly prescribed first-line chemotherapy regimens for HER2-negative or unknown status of GC or GEJC were FOLFOX (5-FU, calcium leucovorin and oxaliplatin), EOX and ECF in the US and in Europe, while TS-1 plus cisplatin and TS-1 plus oxaliplatin were mostly used in Japan [62]. Differences among guidelines have also been observed for HER2 overexpressing tumours. In the US, the most common first-line regimens in 2016 were trastuzumab plus cisplatin plus 5-FU, trastuzumab plus cisplatin plus capecitabine, and FOLFOX, whereas in Western Europe they were trastuzumab plus cisplatin plus 5-FU, and trastuzumab plus cisplatin plus capecitabine. In Japan, the most commonly used regimens in 2015 were trastuzumab plus cisplatin plus capecitabine, trastuzumab plus cisplatin plus TS-1, and TS-1 plus cisplatin (Table S3 in Supplement) [62].

### Humanistic burden of GC and GEJC

No large longitudinal epidemiological studies assessing the humanistic burden of GC from diagnosis to death were identified in our search. Among the identified studies, five were systematic literature reviews [63–67], nine were economic analyses [18, 20, 24, 26, 33, 37, 41, 42, 68], six were randomised controlled trials [69–73], one was an observational study [74], seven were prospective cohort or case-control studies [39, 75–80], seven were cross-sectional survey studies [81–87], four were retrospective studies [88–91] and one included a retrospective analysis and a prospective cross-sectional survey [92]. Only four studies used tools specific to GC or GEJC (GC module [STO22] and oesophago-gastric [OG25] modules of European Organisation for Research and Treatment of Cancer Quality of Life Questionnaire [EORTC-QLQ]) to assess HRQoL [78, 80, 86, 87]. Among all studies reporting humanistic data (*n* = 40), EORTC-QLQ-C30 was the most frequently used cancer-specific tool to assess HRQoL (*n* = 13) [39, 64, 69–72, 74–78, 87, 91]. Symptoms associated with GC and GEJC worsen and change in nature as the disease progresses. Symptoms such as anaemia, loss of appetite, dysphagia, dyspepsia, reflux and insomnia are often reported at any stage, whereas weight loss, abdominal pain, vomiting, gastric obstruction and bleeding are commonly associated with advanced stages. In addition to symptoms that are attributed to the disease, other symptoms are chemotherapy-related [63–65] (Table 1).

Assessments based on the EORTC-QLQ-C30 revealed that patients with GC have worse general well-being, functional difficulties and symptoms than patients with colon and rectal cancer [75] and worse emotional and cognitive functioning than patients with oesophageal cancer [77]. Moreover, patients with GC have worse nausea, vomiting and constipation but comparable fatigue, pain, dyspnoea, sleep disturbance, appetite loss, diarrhoea and financial difficulty versus patients with oesophageal cancer [77]. Assessments conducted at different disease stages show that physical, emotional and social functioning, global HRQoL, fatigue, appetite, weight loss, dyspnoea and constipation worsen as the disease progresses [77].

Assessments of the impact of surgery on HRQoL showed that the negative impact of total or subtotal gastrectomy on various functional scales was completely recovered between 3–6 months after surgery while gastrointestinal symptoms (dietary restrictions, loss of appetite and diarrhoea) persisted longer [64, 87].

Several studies reported the impact of palliative chemotherapy on the HRQoL of patients with advanced GC [69–72, 77]. As GC is associated with rapid disease progression at the advanced stages, the delay of deterioration observed with chemotherapy accounts for the improvement in HRQoL when compared with best supportive care (BSC). At the end of a 16-week chemotherapy treatment, 55% of patients with advanced GC reported a large or moderate improvement in the global HRQoL score compared with baseline, while 19% reported a decrease [72]. Increased scores were also observed in 40%, 60% and 30% of patients in the physical, emotional and social functioning scales, respectively.

Most studies reported the impact of specific chemotherapy regimens on HRQoL. The addition of docetaxel to cisplatin plus 5-FU prolonged the time to 5% worsening of global HRQoL deterioration, 5% physical and social functioning deterioration, 10%, 20% and 30% nausea, vomiting and pain deterioration, respectively, and 30% appetite loss deterioration [69]. Leucovorin plus 5-FU (LV5FU2) plus irinotecan was associated with improved global health compared with LV5FU2 monotherapy or with cisplatin [70].

One study compared the accuracy of the oesophageal cancer-specific scale, EORTC-QLQ-OG25, with the GC-specific scale, EORTC-QLQ-STO22, for the assessment of the HRQoL in patients who underwent partial and total gastrectomy and found that the OG25 was more sensitive than the STO22. In this study, differences between the total and partial gastrectomy groups in weight loss, odynophagia, choking when swallowing and difficulty eating were identified only with the OG25 scale [80], and the OG25 scores for body image, dysphagia, odynophagia, pain and discomfort, anxiety and weight loss were worse after total compared with partial gastrectomy [80].

A study assessing patient satisfaction with different types of surgery and therapies showed that subtotal gastrectomy was associated with higher satisfaction than total gastrectomy and, among different therapies, patient satisfaction was higher for radiotherapy alone than for chemotherapy alone or combination chemotherapy/radiotherapy [84]. Moreover, patients involved in decision making reported higher treatment satisfaction and would or did choose the same treatment again [84]. In patients with advanced GC, the most important objectives of treatment were improving survival (54.6%), avoiding disease progression (34.6%) and having no limitations in daily routine (27.3%) [83].

### Economic burden of GC and GEJC

A total of 28 studies reported costs associated with the management of GC or GEJC [18–45]. A review of the economic data available on GC management shows that, even though GC is a major clinical and financial burden, only a few evaluations of the costs of GC management and cost-effectiveness were available, most of which were conducted in Asia [18–21, 25, 27, 28, 31, 35, 38, 44, 88, 89, 93, 94]. Moreover, few studies were conducted according to high-quality standards and the reported data are not always clear [30]. The cost of GC management per patient is generally higher than the cost for other cancers [27, 28, 36]. The overall healthcare cost of GC compared with other cancers in China [28], Taiwan [27] and the US [36] is reported below (Table 2).

**Figure 3.**
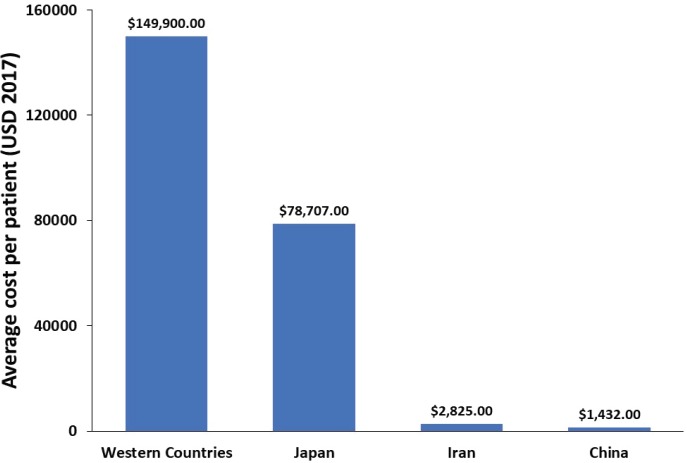
Average overall healthcare cost per GC and GEJC patient.

In Japan, the ASIR of GC has been decreasing in the last 30 years; however, an increase in the number of new GC patients has been reported during the same period due to the rapid population ageing [95]. The direct cost of GC management in Japan has increased between 1996 and 2008 and is predicted to continue to increase until 2020, whereas the total cost is expected to decrease (when considering constant mortality rate) [21]. In Portugal, the total cost of hospitalisation per patient for GC and oesophageal cancer between 2000 and 2010 was €6,422 and €5,049, respectively [32]. The average financial burden per patient was not reported in any of the examined studies. Therefore, using the data provided in the reviewed studies, we estimated a financial burden per patient associated with GC and GEJC in Western countries, Japan, Iran and China (Figure 3).

Furthermore, we estimated the annual financial burden associated with GC for different countries based on the 2012 incidence data and the average healthcare cost per patient (Table 3). Overall, these data reveal a disparity in the cost of GC and GEJC among different regions.

**Figure 4.**
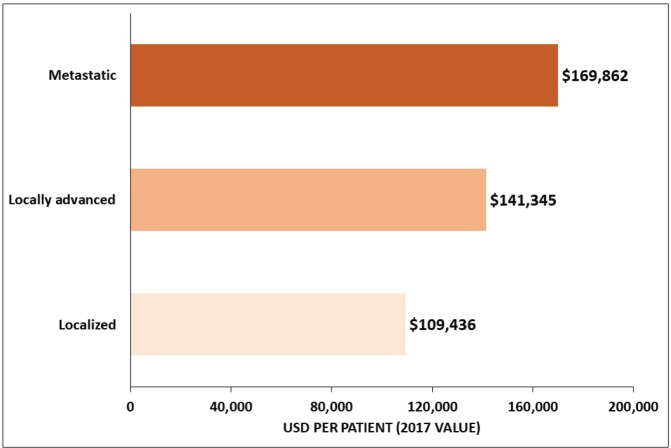
Average overall healthcare cost of GC by tumour stage for ≥65-year-old patients in the US. Source: Yabroff 2008 [36].

Among studies conducted in the US, one of the studies reported an average overall cost of USD 96,571 for a GC patient in the 18 months after diagnosis, which was estimated to be more than 10 times the cost of gender-matched controls [23], whereas the costs of GC per patient ≥65 years old was USD 44,203 for men and USD 41,899 for women over a 5-year period and increased with the tumour stage [36] (Figure 4). Among Medicare-enrolled patients with advanced GC who received first-line treatment, the average total follow-up cost from diagnosis of the advanced or metastatic stage was USD 70,808 per patient [43]. The estimated healthcare costs for GC patients were significantly higher in the US than in Iran, where the average cost per patient was USD 3,940 and USD 2,596 in private and public centres, respectively [25]. Moreover, the cost increased from USD 2,707 for stage I to USD 4,608 for stage IV in private centres, and from USD 2,191 for stage I to USD 2,877 for stage IV in public centres [25].

Several studies examined health resource utilisation. Length of hospital stay in different countries is summarised in Table 4.

The economic burden of GC and GEJC associated with specific treatment modalities was reported in 17 studies in Japan, South Korea, Singapore, China, Sweden, Portugal, the UK and the US. The cost of endoscopic submucosal dissection (ESD) for early GC in Japan and South Korea was evaluated in three studies [31, 44, 94]. Hospitalisation costs were estimated at USD 4,681, USD 5,353 and USD 1,864 for open gastrectomy, laparoscopy-assisted gastrectomy and ESD, respectively, with no differences in the 1-year follow-up costs between ESD and conventional surgeries [94]. In Japan, between 2009 and 2011, the cost of ESD per patient decreased from USD 6,768 to USD 6,428 due to a significant decrease in the length of hospital stay from 10.5 to 9.5 days [31]. The cost of ESD was higher in elderly (≥80 years, USD 7,346) compared with non-elderly patients (<80 years, USD 6,296) due to the longer length of stay in elderly patients (12.2 days versus 9.3 days) [44].

The economic burden of adjuvant and neoadjuvant chemotherapy was reported in four studies [20, 24, 35, 38]. In China, the implementation of adjuvant therapy increased the overall cost compared with gastrectomy alone over the first 3 (USD 17,824 versus USD 9,051) and 5 (USD 23,364 versus USD 20,007) years but decreased the overall cost over 10 (USD 39,889 versus USD 48,284) and 30 (USD 71,537 versus USD 87,004) years. Furthermore, the total direct cost per patient per cycle was lower with adjuvant therapy with S1 (USD 1938 ± 236) than with XELOX (IV oxaliplatin and oral capecitabine, USD 2,317 ± 315) [20]. Similarly, another study showed higher total direct costs per cycle per patient of XELOX (USD 2,317 ± 315) compared with S1 (USD 1,938 ± 236), which was driven by the time cost [38]. A study conducted in Sweden assessed the cost of neoadjuvant systemic chemotherapy followed by cytoreductive surgery and hyperthermic intraperitoneal chemotherapy in peritoneal carcinomatosis from GC compared with systemic chemotherapy alone. The mean costs per patient were higher in the neoadjuvant group (USD 145,728) than in the systemic chemotherapy group (USD 59,314) [24]. A study conducted in Singapore showed that the implementation of a multidisciplinary gastrectomy pathway significantly reduced the mean total (11.29 versus 14.04 days; *P* = 0.023) and post-surgery (8.88 versus 11.00 days; *P* = 0.022) length of hospital stay and average hospitalisation costs per patient (before its implementation: USD 17,371; post-implementation: USD 13,338; *P* = 0.047) [35].

The cost of chemotherapy was assessed in ten studies [18, 19, 22, 26, 29, 33, 41–43, 68]. Two studies compared the costs associated with oral capecitabine versus IV 5-FU in combination with cisplatin as first-line treatment in patients with advanced GC and found that for 5.5 cycles of 21 days each, administration of capecitabine in place of 5-FU resulted in savings of £4210.29 [42] and €5,869 [29]. Two studies conducted in the UK [68] and Japan [33] investigated the economic burden associated with trastuzumab as first-line therapy for HER2-positive advanced GC and reported costs of approximately £26,100 and between €27,000–€30,000, respectively. One study in the UK evaluated the economic burden of ramucirumab either alone or with paclitaxel for advanced GC or GEJC adenocarcinoma previously treated with chemotherapy; total costs were £52,996 for ramucirumab/paclitaxel versus £13,400 for BSC, £18,779 for docetaxel in combination therapy and £36,678 for ramucirumab monotherapy versus £14,137 for BSC monotherapy [41]. A study assessing the cost effectiveness of second-line therapies in the US found that the total lifetime cost per patient ranged between USD 39,264 for irinotecan and USD 143,978 for ramucirumab plus paclitaxel [26]. The annual costs of third- or subsequent-line therapy in GC patients in China amounted to USD 9,915.48 for apatinib and USD 1,801.62 for BSC [18]. Another Chinese study of chemotherapy regimens in advanced GC reported that FAMTX (5-FU, calcium leucovorin, adriamycin and methotrexate) and DCF (docetaxel, cisplatin and 5-FU) were the least (RMB 1,756.95) and most expensive (RMB 9,979), respectively [19]. Total healthcare costs for patients with advanced/unresectable or metastatic GC receiving standard first-line chemotherapy in the US were evaluated in two studies. Between 2000 and 2009, the average costs for first-line, second-line and BSC as first-line were USD 36,810, USD 22,332 and USD 40,628, respectively [43]. Similar results were reported in a retrospective database analysis of chemotherapy treatment patterns and outcomes of patients with GC. Between 2004 and 2012, the costs for first- and second-line treatment were USD 40,810 and USD 26,587, respectively [22].

Based on the data reported in this review, we estimated that the annual financial burden of GC calculated for Europe (including France, Germany, Italy, Spain and the UK), Asia (including Iran, Japan and China), North America (Canada and the US) and Australia amounted to 20.6 billion USD in 2017.

## Discussion

GC is an aggressive cancer that, in 2015, was the third leading cause of cancer-related mortality worldwide and the fifth in incidence [1]. Over the last two decades, a stable decline in the worldwide incidence of non-cardia GC has been observed in both developed and developing countries with marked differences across geographical regions [1, 6, 50, 51]. Possible reasons for this decline in Western countries include the increased availability of fresh fruits and vegetables, decreasing consumption of preserved foods and a decrease in smoking [12]. H. pylori infection has been strongly associated with non-cardia GC, suggesting that the widespread use of antibiotics may have contributed to the decrease in incidence [12]. In contrast, the incidence of cardia GC has increased in the US and several European countries possibly due to the growing incidence of gastroesophageal reflux disease associated with increased obesity [6, 12, 13]. Despite the overall decline in the global incidence of GC, and even if this trend continues, the prevalence of GC is expected to increase in the next decade due to the growing worldwide population. Most countries, particularly those with a low incidence of GC, lack specific screening programmes for GC, and therefore diagnosis often occurs at advanced stages, resulting in a poor prognosis [9–11]. In the past decades, survival rates have significantly improved in various Asian countries where GC screening programmes have been implemented [97], suggesting that efforts to reduce GC rates should focus on early diagnosis. It is likely that control of H. pylori infection and risk factors including reflux disease, obesity, tobacco use and diet would also contribute to decreasing the mortality associated with GC. This is supported by the evidence that in the last 25 years, the rate of oesophageal squamous-cell carcinoma has been declining in several countries, and since this type of oesophageal cancer is associated with alcohol and tobacco use, this decline could be due to a reduction in tobacco and alcohol use [98].

Overall, the treatment guidelines for GC are similar across regions, with surgery and adjuvant chemoradiotherapy reserved for early stages and palliative chemotherapy for later stages, depending on the patient’s health status. Several chemotherapy regimens are available, yet no gold standard exists. In the US, 80% of patients with metastatic GC received first-line chemotherapy in 2016 [62].

The literature search conducted for this review did not identify any large longitudinal epidemiological studies that assessed the humanistic burden of GC. Based on the available data, GC patients have worse general well-being, functional difficulties and symptoms than patients with colon or rectal cancer [75], and disease progression is associated with lower HRQoL [77]. Total or subtotal gastrectomy was found to have a negative impact on physical, cognitive and role functioning, all of which gradually recover between 3 months [64, 87] and 5 years after surgery [78]. Chemotherapy was associated with improvement in the global HRQoL, and physical, emotional and social functioning scores compared with baseline in 30%─60% of patients [72].

Few high-quality studies assessing the economic burden of GC and GEJC were identified. Since most economic evaluations were conducted in Asia, where GC incidence is high and early diagnosis and treatment are more common, it is difficult to extrapolate the conclusions of these studies to other regions. Compared with other cancers, GC is associated with one of the highest economic burdens, with marked differences across different geographic regions. This burden increases as disease progresses, and the highest costs are associated with metastatic tumours. No studies were identified that reported the total cost per patient or the overall financial burden associated with GC in different regions. In Japan, the direct cost associated with GC has increased from 1996 to 2008 and is expected to continue growing until 2020 [21]. In the US, the 5-year cost of care for the elderly Medicare cancer patients diagnosed in 2004 was estimated at $624 million [36], and the healthcare costs incurred by GC patients in the US have been estimated to be at least 10 times those for non-cancer US subjects [23]. We have attempted to estimate the global costs of GC/GEJC based on published cost data and estimated prevalence in different countries. However, since most of the available data are not up-to-date (2015 or earlier), our estimate may not reflect the current global economic burden. This may also explain the significantly lower cost reported for China [28] compared with other countries despite the high GC/GEJC prevalence observed in this country. It is also important to consider that, even though the economic costs associated with GC/GEJC are largely affected by disease stage, this information was not always reported in the reviewed articles. Our calculated cost for the US was lower than the one estimated for Japan. Our estimate for the US was based on an average patient cost calculated across all disease stages and may, therefore, represent a conservative estimate. It is likely that this cost would increase if calculated based on the cost associated with advanced stages of the tumour.

## Conclusion

GC represents a substantial burden to patients, associated with severe symptoms and limited availability of effective treatments, and with economic costs that are expected to continue growing worldwide in the next decade. There is still significant room for improvement with regard to early detection and intervention and the introduction of new life-extending therapies. Moreover, the use of predictive biomarkers to optimise patient selection for specific therapies may improve treatment efficiency and patient outcome.

## List of Abbreviations

5-FU: 5-Fluorouracil
ASIR: Age standardised-incidence rate
BSC: Best supportive care
DCF: Docetaxel, cisplatin and 5-FU
EBV: Epstein-Barr virus
ECF: Epirubicin, cisplatin and fluorouracil
EORTC-QLQ: European Organisation for Research and Treatment of Cancer Quality of Life Questionnaire
EOX: Epirubicin, oxaliplatin and capecitabine
EPIC: Early post-operative intraperitoneal chemotherapy
ESD: Endoscopic submucosal dissection
FAMTX: 5-FU, calcium leucovorin, adriamycin and methotrexate
FOLFOX: 5-FU, calcium leucovorin and oxaliplatin
GC: Gastric cancer
GEJ: Gastroesophageal junction
GEJC: Gastroesophageal junction cancer
HIPEC: Hyperthermic intraperitoneal chemotherapy
HRQoL: Health-related quality of life
HTA: Health Technology Assessment
LV5FU2: Leucovorin plus 5-FU
OG25: Oesophago-gastric
PD-1: Programmed death receptor 1
SD: Standard deviation
STO22: Gastric cancer module
USD: United States dollar
XELOX: IV oxaliplatin and oral capecitabine

## Conflicts of Interest

M Casamayor reports personal fees from Astellas during the conduct of the study. R Morlock and H Maeda are employed by Astellas. J Ajani has no relevant conflict of interest to declare.

## Figures and Tables

**Table S1.** Recommended first-line regimens for advanced GC in Europe, US and Japan.

Therapy	ESMO	NCCN	JGCA
Preferred	Capecitabine + platinum (cisplatin or oxaliplatin) [IA]	Fluoropyrimidine (5-FU or capecitabine) + cisplatin [I] Fluoropyrimidine (5-FU or capecitabine) + oxaliplatin [2A]	S-1 + cisplatin [I]
Other	Fluoropyrimidine (5-FU or capecitabine) + platinum (cisplatin or oxaliplatin) [IA] Anthracycline-based triplets: (ECF, ECX, EOF, EOX) Triplets containing taxanes: DCF [I, C]	Paclitaxel + cisplatin or carboplatin [2A] Docetaxel + cisplatin [2A] 5-FU or capecitabine [2A] Docetaxel [2A] Paclitaxel [2A] 5-FU + irinotecan [1] DCF [2A] DOF [2A] DF + carboplatin [2B] ECF [I] EOF [I] EOX [I] ECX [I]	Capecitabine + cisplatin [2] S-1 + docetaxel [2] Irinotecan + cisplatin [3 – not recommended] S-1 + irinotecan [3] 5-FU + cisplatin + docetaxel [3] S-1 + oxaliplatin [2] Capecitabine + oxaliplatin [2]


Sources: Ajani 2016; JGCA 2017; Smyth 2016

Abbreviations: DCF = docetaxel, cisplatin and 5-fluorouracil, DF = docetaxel and 5-fluorouracil, DOF = docetaxel, oxaliplatin and 5-fluorouracil, ECF = epirubicin, cisplatin and fluorouracil, ECX = epirubicin, cisplatin and capecitabine, EOF = epirubicin, oxaliplatin and 5-fluorouracil, EOX = epirubicin, oxaliplatin and capecitabine

**Table S2.** Key recommendations for first- and second-line treatment of advanced and metastatic GC and GEJC.

Reference (Association)	Recommendation for advanced and metastatic GC and GEJC (First-line treatment)	Recommendations for advanced and metastatic HER2-positive GC and GEJC (Second- and subsequent lines of treatment)
**Italian Medical Oncology Association, 2016 [1]**	Chemotherapy in asymptomatic patients Combined therapy Triplet chemotherapy in patients with good health Oral fluoropyrimidine can substitute 5-FU Oxaliplatin can substitute cisplatin Irinotecan plus fluorouracil Fluoropyrimidine + cisplatin + trastuzumab (If HER2-positive overexpression) Oxaliplatin + 5-FU Capecitabine can replace 5-FU Irinotecan + 5-FU	Second-line therapy Should be considered if a patient who has progressed after first-line is in good condition and has a PS of 0 or 1 [high; strong positive] Combined therapy with ramucirumab + paclitaxel is currently considered the SOC If not eligible for paclitaxel, monotherapy with ramucirumab
**National Comprehensive Cancer Network, 2016 [2]**	Two-drug cytotoxic regimens Three-drug cytotoxic regimens for medically fit patients Preferred Regimens Fluoropyrimidine (fluorouracil or capecitabine) and cisplatin Fluoropyrimidine (fluorouracil or capecitabine) and oxaliplatin Other Regimens Paclitaxel with cisplatin or carboplatin; docetaxel with cisplatin; fluoropyrimidine (fluorouracil or capecitabine); docetaxel; paclitaxel; fluorouracil and irinotecan [category 1]; DCF modifications: docetaxel, cisplatin and fluorouracil; docetaxel, oxaliplatin and fluorouracil; docetaxel, carboplatin and fluorouracil [category 2B]; ECF (epirubicin, cisplatin and fluorouracil) [category 1]; ECF modifications [category 1]: epirubicin, oxaliplatin and fluorouracil; epirubicin, cisplatin and capecitabine; epirubicin, oxaliplatin and capecitabine	Preferred second-line regimens Ramucirumab and paclitaxel [category 1]; docetaxel [category 1]; paclitaxel [category 1]; irinotecan [category 1]; ramucirumab [category 1] Other second-line regimens Irinotecan and cisplatin; fluoropyrimidine (fluorouracil or capecitabine) and irinotecan [category 2B]; docetaxel and irinotecan [category 2B]
**Japanese Gastric Cancer Association, 2014 [3]**	Chemotherapy for unresectable/recurrent GC, after non-curative R2 resection, if PS 0–2, with the unresectable T4b disease, extensive nodal disease, hepatic metastases, peritoneal dissemination or other M1 disease First-line therapies (HER2-negative) S-1 + cisplatin combination is the SOC Capecitabine + cisplatin combination S-1 + docetaxel combination (for outpatient treatment) Irinotecan + cisplatin, S-1 + irinotecan and 5-FU + cisplatin + docetaxel not recommended S-1 + cisplatin + docetaxel (DCS regimen) under evaluation S-1 + oxaliplatin [category 2] Capecitabine + oxaliplatin [category 2] First-line therapies (HER2-positive) Trastuzumab + cisplatin + either capecitabine or infusional 5-FU [category 1] Trastuzumab + S-1 + cisplatin [category 2]	Second-line treatment Recommended for patients with sufficient PS Docetaxel monotherapy [category 1] Irinotecan monotherapy [category 1] Paclitaxel (weekly administration) monotherapy [category 1] Paclitaxel + ramucirumab [category 1] Ramucirumab monotherapy [category 2]
**National Institute for Health and Care Excellence. Stomach cancer, 2017 [4]**	First-line treatment of inoperable advanced GC Capecitabine and a platinum-based regimen My5-FU assay only recommended for guiding dose adjustment in people having fluorouracil chemotherapy by continuous infusion.	Ramucirumab alone or with paclitaxel is not recommended within its marketing authorisation for advanced GC or GEJ adenocarcinoma previously treated with chemotherapy
**Spanish Society of Medical Oncology, 2015 [5]**	First-line treatment Palliative chemotherapy increases median OS and improves the HRQoL of patients with advanced GC [I,A] Both CF and ECF can be considered standard combinations [IA] Triplets with docetaxel can be considered in selected patients [IB] Oxaliplatin has similar efficacy and less toxicity than cisplatin and can replace it in this setting [IA] Oral fluoropyrimidines (capecitabine or S-1) can replace 5-FU in this setting [I,A] Irinotecan combinations (FOLFIRI, IF) have similar efficacy and better tolerance than CF and can be considered adequate options for these patients [I,B] Trastuzumab added to cisplatin + fluoropyrimidines can be considered the standard treatment in patients with gastric or GEJ advanced HER2-positive adenocarcinoma [IB]	Second-line chemotherapy <60 % of patients receive second- or third-line therapy for GC in clinical practice Second-line chemotherapy achieves improvement in HRQoL and provides a median OS of 4–6 months [I,A] Docetaxel, paclitaxel, irinotecan, and a targeted therapy against VEGFR2 with ramucirumab have demonstrated a significant benefit in OS in phase III trials [I,B] and a significant reduction in the risk of death [I,A] Irinotecan has shown a statistically significant survival benefit over BSC (median OS 4 versus 2.4 months, respectively) [I,B] Ramucirumab has shown a significant OS benefit (5.2 months versus 3.8 months, HR = 0.77) over placebo [I,B] Ramucirumab plus paclitaxel arm showed a significantly superior OS (9.6 months versus 7.3 months, HR = 0.80) over paclitaxel monotherapy [I,B]
**European Society for Medical Oncology, 2016 [6]**	First-line treatment Doublet or triplet platinum/fluoropyrimidine for fit patients with advanced GC Chemotherapy is recommended for patients with inoperable locally advanced and/or metastatic (stage IV) disease [I, A]. Comorbidities, organ function, and PS must always be taken into consideration [II, B] Capecitabine is associated with improved OS compared with infused 5-FU within doublet and triplet regimens [I, A] DCF in a 3-weekly regimen was associated with improved OS, but also added significant toxic effects including increased rates of febrile neutropenia [I, C] Elderly patients with GC Capecitabine and oxaliplatin, FOLFOX, single-agent capecitabine and S1 (in Asian patients) [III, B] The FLOT regimen was associated with a trend towards improved PFS but also with increased toxicity [II, B] Metastasised patients Gastrectomy in patients with the limited metastatic disease does not improve survival [I, A] The use of cytoreductive surgery plus HIPEC cannot yet be recommended in patients with peritoneal metastases	Second-line chemotherapy with a taxane (docetaxel, paclitaxel), or irinotecan, or ramucirumab as a single agent or in combination with paclitaxel is recommended for patients who are of PS 0–1 [I, A] Similar efficacy has been demonstrated for weekly paclitaxel and irinotecan [I, A] In patients with disease progression >3 months following first-line chemotherapy, it may be appropriate to consider a rechallenge with the same drug combination [IV, C] In patients with symptomatic locally advanced or recurrent disease, hypofractionated RT is an effective and well-tolerated treatment modality that may palliate bleeding, obstructive symptoms or pain [III, B]

Abbreviations: 5-FU = 5-fluorouracil, BSC = best supportive care, CF = cisplatin plus 5-FU, DCF = docetaxel, cisplatin and 5-FU, DCS = S-1, cisplatin and docetaxel, ECF = epirubicin, cisplatin and fluorouracil, FLOT = fluorouracil, leucovorin, oxaliplatin and docetaxel, FOLFIRI = folinic acid, 5-FU and irinotecan, FOLFOX = 5-FU, calcium leucovorin and oxaliplatin, GC = gastric cancer, GEJ = gastroesophageal junction, GEJC = gastroesophageal junction cancer, HER2 = human epidermal growth factor receptor 2, HIPEC = hyperthermic intraperitoneal chemotherapy, HR = hazard ratio, HRQoL = health-related quality of life, IF = irinotecan and fluorouracil, OS = overall survival, PFS = progression-free survival, PS = performance status, RT = radiotherapy, SOC = standard of care, VEGFR2 = vascular endothelial growth factor receptor 2

**Table S3.** Most prescribed chemotherapy regimens in Western Europe, the US and Japan.

	Western Europe (2016)	US (2016)	Japan (2015)
**HER2-negative**			
First-line	1. FOLFOX 2. EOX 3. ECF	1. FOLFOX 2. EOX 3. ECF	1. TS-1 + cisplatin 2. TS-1 + oxaliplatin
Second-line	1. Docetaxel 2. FOLFIRI 3. Ramucirumab + paclitaxel	1. Ramucirumab + paclitaxel 2. FOLFIRI 3. FOLFOX	1. Paclitaxel 2. Irinotecan 3. Ramucirumab
Third-line	1. Irinotecan 2. FOLFIRI 3. Docetaxel	1. Ramucirumab + paclitaxel 2. Ramucirumab 3. FOLFIRI	1. Irinotecan 2. Paclitaxel 3. Abraxane
**HER2 overexpressing**			
First-line	1. HCF 2. HCX	1. HCF 2. HCX 3. FOLFOX	1. HCX 2. HC + TS-1 3. TS-1 + cisplatin

Abbreviations: FOLFIRI = folinic acid, 5-FU and irinotecan, FOLFOX = 5-FU, calcium leucovorin and oxaliplatin, EOX = epirubicin, oxaliplatin and capecitabine, ECF = epirubicin, cisplatin and fluorouracil, HCF = trastuzumab, cisplatin and 5-FU, HCX = trastuzumab, cisplatin and capecitabine, HER2 = human epidermal growth factor receptor 2

**Table 1.** Common symptoms associated with advanced GC and chemotherapy.

Symptom	Disease-related symptoms	Chemotherapy-related symptoms
Abdominal pain [39, 69, 86, 88, 89]	25–62	3–24
Indigestion [86]	60	
Fatigue [39, 86]	58	5–55
Constipation [86]	40	
Weight loss [69]	25–45	
Nausea/vomiting [39, 88, 89]	10–33	10–55
Gastric obstruction [88, 89]	5–31	
Ascites [88]	3–35	
Bleeding [88, 89]	2–24	
Diarrhoea [39]		4–44

Data are presented as proportion of patients

**Table 2.** Total annual healthcare cost of GC per patient for different cancers in different regions.

Cancer Location	USD (2017 value)		
	Taiwan (2007)^#^	China (2013)	USA (1998–2003)*^#^
Gastric	11,997	1,486	46,501
Breast	9,290	–	–
Colorectal	9,029	1,683	29,609
Liver	8,177	–	41,284
Lung	11,887	–	35,672
Oesophageal	–	2,135	49,811

# Cost relative to the first 12 months after diagnosis; net cost in 2004 USD relative to men only

**Table 3.** Annual cost of GC and GEJC in different geographic regions (2017 value).

Geographic Region	Country	Annual Cost*(Million USD)
Europe	France	975
	Germany	2,401
	Italy	1,949
	Spain	1,171
	UK	1,002
North America	Canada	501
	US	3,171
Asia	Iran	27
	Japan	8,492
	China	580
Oceania	Australia	366

* Based on incidence of GC in 2012 [7] and an average cost (US dollars, 2017 values) per patient of $149,900 in Western countries [36], $78,707 in Japan [21], $2,825 in Iran [25] and $1,432 in China [28]

**Table 4.** Mean length of hospitalisation for GC patients.

Country	Mean Length of Hospital Stay per Patient by Treatment (days)
**Asia**	
China [28]	Median, 9
Japan [44]	Overall (elderly): 12.2 Overall (non-elderly): 9.3
Japan [31]	Overall (2009): 10.5 Overall (2010): 9.8 Overall (2011): 9.5
Singapore [35]	Pathway group*: 11.29 Pre-pathway group**: 14.04
South Korea [88]	1st line: 8.2 (SD, 9.4) 2nd line: 9.1 (SD, 11.3) BSC after 1st line: 14.3 (SD, 15.6) 3rd line: 10.2 (SD, 12.5)
South Korea [93]	Overall: 8.7 (SD, 9.8)
Taiwan [89, 93]	1st line: 6.8 (SD, 8.5) BSC after 1st line: 22.1 (SD, 19.9) 2nd line: 8.8 (SD, 10.1) Overall: 8.6 (SD, 10.0)
**Europe**	
Portugal [32]	Median (between 2000–2010), 11
Sweden [24]	Cytoreduction plus HIPEC plus EPIC: 57 (range, 13–215) Systemic chemotherapy: 26 (range, 0–46)
**North America**	
Canada [96]	Overall: 30 (SD, 30)
US [43]	Overall: 8 (SD, 7) Additionally treated after 1st line^#^ All post–first-line: 7 (SD, 6) First-line: 9 (SD, 8) Second-line: 7 (SD, 6) Supportive care only All post–first-line: 8 (SD, 7) First-line: 9 (SD, 7)
US [23]	Overall: 22.1

# Patients receiving additional cancer-related treatment after first-line chemotherapy

* Patients managed according to a multidisciplinary clinical programme

** Patients managed conventionally
